# Innate lymphoid cells in inflammatory arthritis

**DOI:** 10.1186/s13075-020-2115-4

**Published:** 2020-02-12

**Authors:** Weiting Fang, Yuanyuan Zhang, Zhu Chen

**Affiliations:** 1 Department of Rheumatology and Immunology, Anhui Medical University Affiliated Provincial Hospital, Lujiang Str 17, Hefei, 230001 China; 2 Department of Rheumatology and Immunology, Division of Life Sciences and Medicine, The First Affiliated Hospital of USTC, University of Science and Technology of China, Lujiang Str 17, Hefei, 230001 China

**Keywords:** Innate lymphoid cells, Rheumatoid arthritis, Spondyloarthritis

## Abstract

Aberrant activation and dysregulation of immune system is a common feature of many forms of inflammatory arthritis. Since their identification as a distinctive population of leukocytes, innate lymphoid cells (ILCs) have been considered crucial in maintaining tissue homeostasis and bridges between innate and adaptive immune system. Altered ILCs’ subset distribution and function have been observed in a variety of autoimmune and chronic inflammatory diseases and suggest a subset-specific role of ILCs in the pathogenesis of immune-mediated inflammation. In this review, we focus on the current knowledge of ILC subset and their role in inflammatory arthritis, including rheumatoid arthritis (RA), ankylosing spondylitis (AS), psoriatic arthritis (PsA), enteropathic arthritis, and other seronegative spondyloarthritis. By better understanding the biology and function of ILC subset in different disease settings, new therapeutic interventions can be anticipated by modulating dysregulated ILC responses toward promoting resolution of inflammation.

## Introduction

Inflammatory arthritis encompasses a group of diseases characterized by chronic inflammation in the joints and adjacent tissues. These include rheumatoid arthritis (RA), ankylosing spondylitis (AS), psoriatic arthritis (PsA), juvenile idiopathic arthritis (JIA), and other seronegative spondyloarthritis. Although the pathogenesis remains unclear, many forms of inflammatory arthritis have autoimmune features, where activated T cells and B cells form a complex network, promoting the production of autoantibodies and contributing to the persistent joint damage [1, 2]. Thus, conventional concepts have strongly been focused on the dysregulated adaptive immune system. However, it has come to an attention that innate immune system also plays a pivotal role in the pathogenesis of inflammatory arthritis.

Innate lymphoid cells (ILCs) are recently identified important effector cells of innate immune system which are distinct from conventional lymphoid lineages. Characterized by the absence of recombination activating gene (RAG)-dependent rearranged antigen-specific receptors, ILCs are mainly located at barrier surfaces in the body, responded promptly to the environmental stress signals, and involved in protection against pathogens, tissue remodeling, and maintenance of homeostasis [3]. Thus, dysregulated activation of ILCs has been linked to the pathogenesis of many inflammatory and autoimmune diseases such as multiple sclerosis, inflammatory bowel disease, systemic sclerosis, and anti-neutrophil cytoplasmic antibody (ANCA)-associated vasculitis as well as RA [4–8].

## ILC subsets

So far, three different ILC subsets have been identified on the basis of transcription factor expression and cytokine production, which resemble from T cell counterpart: group 1 ILCs (comprise conventional NK cells and ILC1s), group 2 ILCs (ILC2s), and group 3 ILCs (consist of ILC3s and LTi cells). With the advanced understanding of their development and function, it has been recently proposed to classify ILCs into five subsets—NK cells, ILC1s, ILC2s, ILC3s, and lymphoid tissue-inducer (LTi) cells, which was approved by the International Union of Immunological Societies [9].

ILC1s are characterized by the expression of transcription factor T-bet and production of IFN-γ in response to IL-12, IL-15, and IL-18 to fight with intracellular pathogens [3]. Although share many features of NK cells such as natural killer cell p46-related protein (NKp46) and NK1.1, it has been increasingly clear that ILC1s were developmentally and functionally distinct from conventional NK cells. ILC1s are tissue resident and have less cytotoxic activity whereas NK cells can account for up to 15% of blood lymphocyte in circulation and exert cytotoxic activity via secretion of perforin and granzyme B [10]. Indeed, ILC1s are heterogeneous and differ in phenotypic markers depending on the tissue microenvironment. For example, small intestine intraepithelial ILC1s express CD160 and require NFIL3 and T-bet for development [11]. In contrast, ILC1s in salivary gland express integrin CD103, CD39, and TNF-related apoptosis-inducing ligand (TRAIL) but with less IFN-γ production. Furthermore, they uniquely require T-bet and Eomes for development without NFIL3 [12].

ILC2s are defined by production of type 2 cytokines such as IL-4, IL-5, and IL-13. They respond to epithelial cell-derived alarmins IL-25, IL-33, and thymic stromal lymphopoietin (TSLP), which in turn launch Th2 responses to expel helminth infection and involved in allergic airway inflammation [3]. Recent studies have demonstrated that ILC2s were rapidly proliferated and activated to initiate Th2 responses by cholinergic neurons-derived neuropeptide Neuromedin U [13–15]. Consistent with Th2 cells, ILC2s require transcription factor GATA3 for development, maintenance, and function [16]. ILC2s are also heterogeneous, and two distinct subsets of ILC2s, i.e., natural ILC2s and inflammatory ILC2s which respond to IL-33 and IL-25, respectively, have been reported [17].

ILC3s are found abundant at mucosal sites and implicated in the maintenance of intestinal homeostasis. Similar to Th17 cells, ILC3s require transcription factor RORγt for development and function, characterized by production of IL-17A, IL-22, and granulocyte-macrophage colony-stimulating factor (GM-CSF) [18]. Two subsets of ILC3s have been identified based on the surface expression of natural cytotoxicity receptor (NCR), in which NCR^+^ ILC3s express NKp46 (in mice) or NKp44 (in humans) and produce high amount of IFN-γ [19].

Like ILC3s, LTi cells are also strictly dependent on RORγt for development and now believed to constitute a subpopulation of ILC3s [18]. They express high amount of CCR6 and are crucial for the development of secondary lymphoid organs during embryogenesis. The main characteristics of the five ILC subsets are summarized in Table 1.

**Table 1:** Main characteristics of ILC subsets

ILC subsets	Surface markers	Transcription factors	Effector mediators	Immune function
NK	NCR, NK1.1, KLRG1, CD122, NKG2D	T-bet, Eomes, NFIL3	Perforin, granzyme B	Fight with intracellular pathogens like virus; anti-tumor immunity
ILC1	NCR, NK1.1, TRAIL, CD200R, CD122, CD160, CD103, CD39	T-bet, Eomes, NFIL3	IFN-γ	Defense against intracellular pathogens, such as virus and protozoan
ILC2	CD127, ST2, ICOS, CRTH2, MHC-II, Sca-1	Gata3	IL-4, IL-5, IL-9, IL-13	Expel extracellular parasites and involve in allergic airway inflammation
ILC3	NCR, RANKL, IL-23R, CD49d, MHC-II	RORγt, T-bet	IL-17, IL-22, GM-CSF	Combat extracellular microbes, such as bacteria and fungi
LTi	CD127, CD117, CCR6, RANKL, IL-23R, MHC-II	RORγt	IL-17, IL-22, TNF, lymphotoxin	Formation of secondary lymphoid structures

### ILCs in homeostasis and chronic inflammation

Since their identification as separate innate immune populations 10 years ago, ILCs have extended our perception of immune regulation and how the immune system contributes to the maintenance of tissue homeostasis. Although there is a lack of pattern-recognition receptors, ILCs are highly sensitive to environmental injury signals for activation. Serving as tissue resident “sentinel” cells, ILCs react promptly to pathogens by sensing epithelial-derived cytokines, alarmins, and inflammatory mediators such as IL-12 for NK and ILC1s; IL-25, IL-33, TSLP for ILC2s; and IL-23 for ILC3s [3], thereby limiting invading pathogens spread and restoring tissue homeostasis. In inflammatory disease settings, accumulating evidence has suggested that activated ILCs in the local tissue were important source of inflammatory cytokines. For example, a recent research has reported that ILCs in the gut express toll-like receptor 2 (TLR2), TLR3, and TLR9, which respond to respective TLR ligands with enhanced production of TNFα and IFN-γ, thus contributing to gut damage [20]. ILC activation is also regulated by a balance of activating and inhibitory signals forwarded by several surface markers. For example, ICOSL which is expressed on ILC2s binds with ICOS to promote ILC2 proliferation and activation. In contrast, the interaction of KLRG1 and E-cadherin has been shown to inhibit ILC2s [21]. The mechanisms of how ILCs were regulated in inflammation and tissue homeostasis have been reviewed recently elsewhere [22].

In addition, emerging evidence suggest that ILCs serve as antigen-presenting cells to initiate helper T cell responses [23]. Thus, the interaction between ILCs and T cells plays an essential role in mounting the most appropriate immune responses against the threat encountered by the individual to maintain homeostasis. However, chronic exposure to environmental stimuli will shift a tissue-protective response into chronic inflammation and pathological injury, in which ILCs were dysregulated or overreacted to some extent [24]. In recent years, it has been reported that ILCs were involved in the pathogenesis of many rheumatic diseases, most of which are characterized by systemic autoimmune responses and persistent uncontrolled inflammation. In this review, we focus on the current understanding and recent advancement of the role of ILCs in inflammatory arthritis.

## Rheumatoid arthritis

Rheumatoid arthritis (RA) is the most common chronic inflammatory arthritis which affects around 1% of world population. It is generally considered that genetic predisposition and environmental risk factors contribute to the breakdown of immune tolerance, leading to the production of autoantibodies and persistent inflammation in the joints [25]. The pathology of RA was characterized by infiltration of inflammatory cells into synovium, proliferation of pannus, and subsequent erosion of cartilage and bone. Among the effector cells, macrophages are central players in synovitis, which act through releasing of substantial amount of inflammatory cytokines such as TNFα, IL-6, IL-1β, and IL-17, indicating a Th1 and (or) Th17 predominant cytokine milieu in the inflamed joints.

An early study reported a subset of CD3^−^CD56^bright^ NK cells were significantly expanded in synovial fluid of RA patients. These cells responded to combined stimulation of IL-12 and IL-15 by secreting IFN-γ [26]. The authors further found that these cells accumulate in the synovial tissues and produce more IFN-γ than their peripheral blood counterparts and promote TNFα production by CD14^+^ monocytes in the joints under the stimulation with IL-12, IL-15, and IL-18 [27]. Although CD56 was previously recognized as an NK cell marker, it could also be ILC1s in the present view, serving as a substantial origin of proinflammatory cytokines in RA pathogenesis [28]. Indeed, a recent study reported that ILC1s were the most predominant ILCs in the synovial fluid of RA patients, which is distinct from patients with psoriatic arthritis (PsA) [29]. In another study, both ILC1s and ILC3s were expanded in the synovial fluid of patients with juvenile idiopathic arthritis (JIA), which corresponded to an increased expression of T-bet and IFN-γ, whereas only NKp44^−^ ILC3s displayed the strongest positive correlation with clinical features [30].

Since lymph node (LN) activation and production of autoantibodies precede the onset of joint inflammation, one study investigated the frequency and ILC profile in LN biopsy specimens from patients with pre-RA, that is, patients were positive for rheumatoid factor and/or anti-citrullinated protein antibodies but without clinical signs of arthritis [31]. Although there is no difference of total ILC frequency in pre-RA compared with early RA (disease duration < 1 year) and healthy controls, the distribution of ILC subset differed among groups. ILC1s were significantly increased both in pre-RA and early RA patients whereas ILC3s were expanded only in early RA patients [31]. However, the limitation of this study was that they did not distinguish ILC1s from ILC2s, leaving the possibility that those ILC1 population include ILC2s. Interestingly, while fewer ILC3s were present in pre-RA, the population of LTi cells was increased in pre-RA compared with early RA patients, suggesting ILC distribution in LN shift from homeostatic profile toward a more inflammatory ILC3 profile thus providing evidence of a role for ILC3s in RA pathogenesis [31]. Actually, earlier study has shown that NKp44^+^ ILC3-like cells were enriched in the synovial fluid of RA patients and positively correlated with disease activity. In addition, these ILC3-like cells were an innate source of IL-22 and TNFα [32]. Consistently, in a Japanese RA population, the frequency of CCR6^+^ILC3-like cells in synovial fluid was positively correlated with tender joint counts and swollen joint counts [33]. However, conflicting results exist, evidenced by the observation that ILC3s were less predominant than ILC1s in RA synovial fluid [29]. This is consistent with the paucity of ILC3s in RA synovial tertiary lymphoid structures [34]. These discrepancies might be explained by the different gating strategies used among studies as well as different disease stages of RA patients enrolled.

**Fig. 1:**
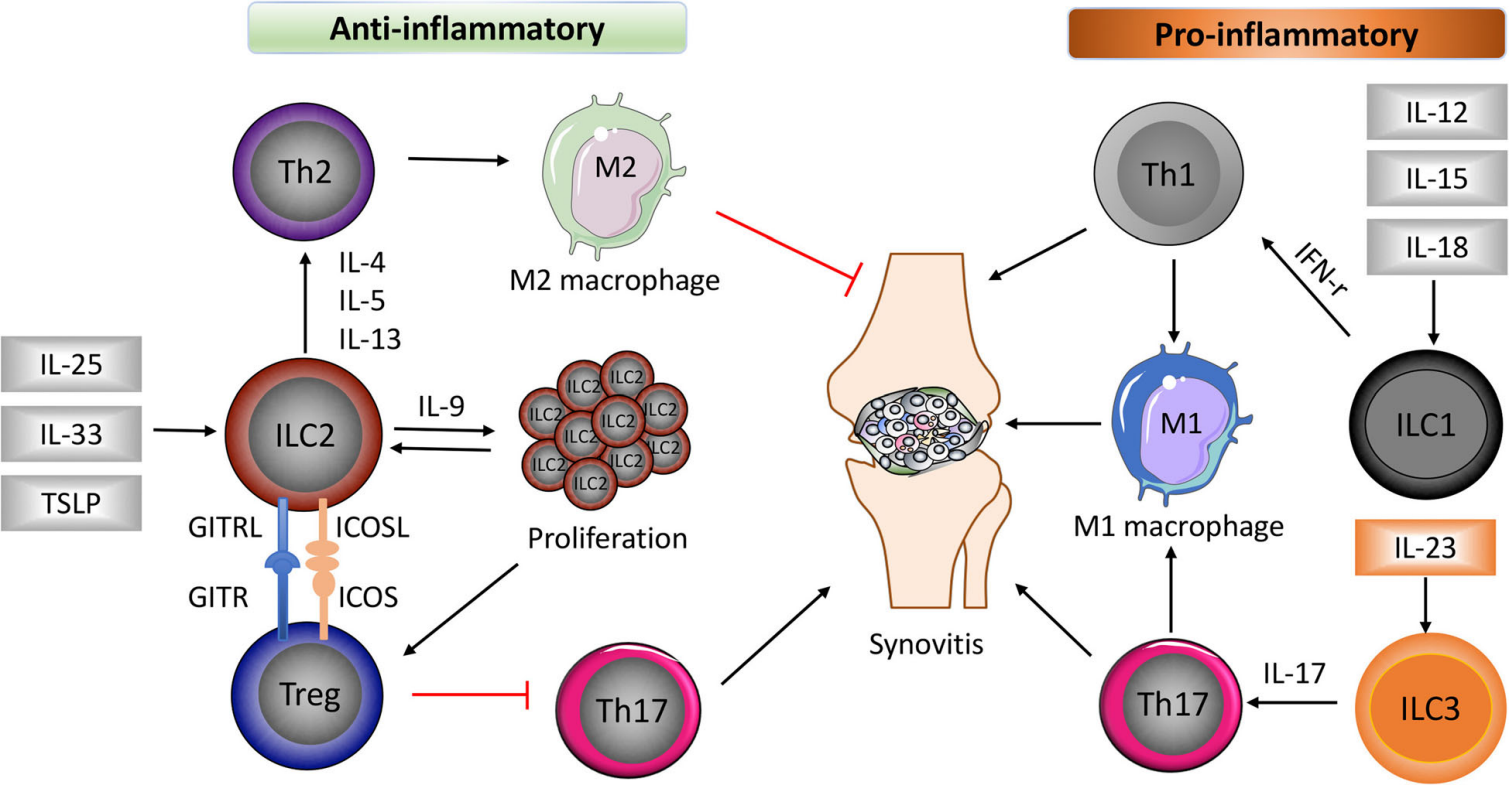
The role of different ILC subsets in RA. Damage in the epithelium cells leads to the release of alarmins such as IL-25, IL-33, and thymic stromal lymphopoietin (TSLP), which strongly induces ILC2 differentiation and subsequent Th2 activation by producing IL-4, IL-5, and IL-13. ILC2s proliferate under IL-9 stimulation and activate regulatory T cells (Treg) via binding of glucocorticoid-induced TNFR-related protein (GITR) ligand (GITRL) to GITR and inducible co-stimulator (ICOS) ligand (ICOSL) to ICOS. Th2 activation induces anti-inflammatory M2 macrophages, together with decreased Th17 responses promote resolution of synovitis. In contrast, ILC1s and ILC3s were differentiated under stimulation of IL-12, IL-15, IL-18, and IL-23, respectively, which further cause Th1 and Th17 activation, proinflammatory M1 macrophage induction, and aggravate inflammation in the joint

In contrast to “proinflammatory” ILC1s/ILC3s, peripheral ILC2s were reduced in active RA patients compared with patients in remission [8]. Moreover, numbers of circulating ILC2s were inversely correlated with disease activity score 28 (DAS28) and increased after receiving anti-inflammatory treatment. In a mouse arthritis model, a reduction of ILC2 accumulation in the arthritic joint of IL-9^−/−^ mice was associated with delayed resolution of arthritis. Overexpression of IL-9 completely reconstituted ILC2s and accelerated the resolution of K/BxN serum-induced arthritis [8]. Mechanistically, ILC2s enhanced regulatory T cells’ (Treg) suppressive function via ICOS-ICOSL and GITR-GITRL interactions, which was required for resolution of joint inflammation [8]. In another recent study, genetic deletion of ILC2s in mice resulted in exacerbated arthritis, whereas increasing ILC2 number by IL-25/IL-33 mini-circles or adoptive transfer significantly attenuated arthritis by affecting the initiation phase [35]. Interestingly, a previous study reported that systemic administration of IL-33 strongly protected against the onset of collagen-induced arthritis, accompanied by induction of Th2 cells, ILC2s, and expansion of Treg [36]. Taken together, all these data suggested an immune-regulatory and anti-inflammatory effect of ILC2s in promoting resolution of chronic inflammation like RA. The general role of ILCs in RA was illustrated in Fig. 1.

## Spondyloarthritis

Spondyloarthritis (SpA) comprises a group of inflammatory diseases including ankylosing spondylitis (AS), reactive arthritis, psoriatic arthritis (PsA), enteropathic arthritis, and undifferentiated spondyloarthritis. They have a strong genetic association with HLA-B27 and share some common clinical features such as spondylitis, peripheral asymmetric arthritis, enthesitis, and extra-articular manifestations. The current concept of SpA is that IL-23/IL-17 cytokine axis was robust drivers of disease, supported by the fact that targeted therapy against IL-17 has been very successful in the treatment of AS and PsA [37, 38]. Since ILC3s have been characterized by production of IL-17, IL-22, and GM-CSF, which is similar to their Th17 counterpart [3, 18, 39], there has been some evidence supporting the involvement of ILC3s in SpA.

**Fig. 2:**
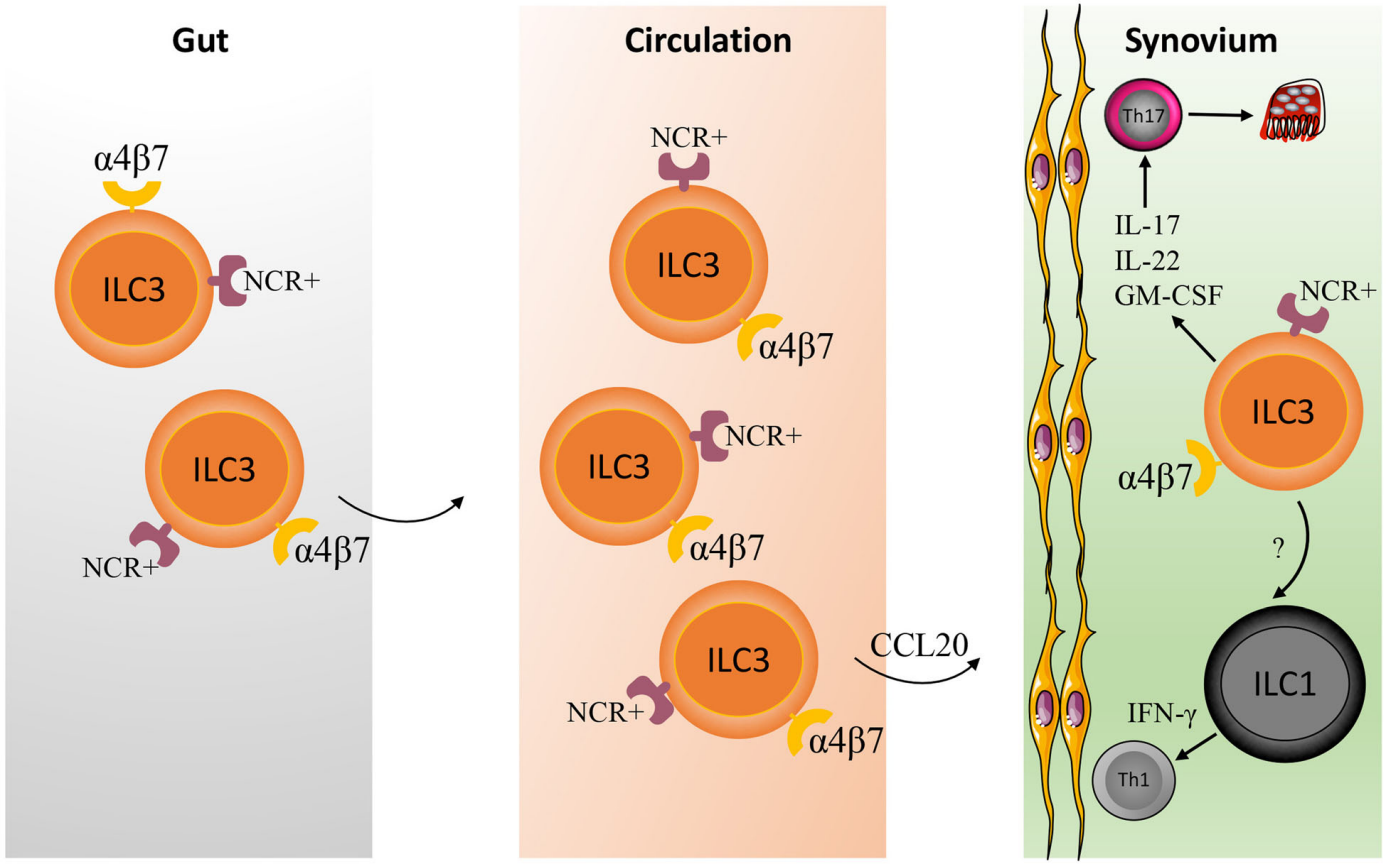
The role of ILC3s in SpA. The intestinal mucosa harbors abundant NCR^+^α4β7^+^ILC3s in homeostatic conditions. In inflammatory settings like SpA, NCR^+^α4β7^+^ILC3s expanded and emigrated from gut into circulation and α4β7-ligand expressing synovial tissue via chemokine like CCL20, where they produce inflammatory cytokines such as IL-17, IL-22, and GM-CSF to promote local joint inflammation. In the meantime, ILC3s might acquire ILC1-like properties under stimulation of IL-12

AS is the most common form of SpA which is categorized into axial SpA and characterized by sacroiliitis, spondylitis, and enthesitis, leading to pathogenic new bone formation and axial ankyloses. It has been described that NKp44^+^ILC3s are present in entheses and perientheseal bone of patients without systemic inflammatory diseases and respond to IL-23/IL-1β by producing IL-17A, indicating the potential role of ILC3s in the pathogenesis of AS [40]. Actually, there have been some studies which demonstrated the enrichment of ILC3s in AS. Ciccia et al. reported that IL-22-producing NKp44^+^ILC3-like cells were increased in the gut of AS patients compared with healthy controls and Crohn’s disease patients [41]. In the follow-up study, the authors found IL-17- and IL-22-producing ILC3s were expanded in the peripheral blood, gut, synovial fluid, and bone marrow of AS patients. These cells have an increased expression of gut-homing integrin α4β7 compared with healthy controls, indicating the possibility that gut-derived ILC3s emigrate from intestine to α4β7 ligand-expressing synovial tissue, promoting local joint inflammation by producing IL-17 and IL-22 [42]. Thereby, it was proposed that ILC3s function as “cytokine shuttles” to travel from gut to extra-intestinal synovial tissues [43] (Fig. 2). Notably, these IL-17/IL-22-producing ILC3s in AS patients appeared to be RORC^−^ but T-bet^+^ thus close to the phenotype of ILC1s, which might reflect the plasticity of ILCs [42]. In addition, GM-CSF-producing ILC3s were also found to be expanded in the synovial tissue of SpA patients [44]. Consistently, a very recent study confirmed NKp44^+^ILC3s were accumulated in the inflamed joints of peripheral SpA patients [45]. Although expression of Th17 signature transcripts such as RORC, AHR, and IL-23R were identified in a large proportion of synovial tissue ILC3s, these cells produce IL-22 and GM-CSF but not IL-17, suggesting they are not a significant source of IL-17 in SpA [45].

In line with the pathogenic role of ILC3s in AS, enrichment of ILC3s in the synovial fluid of inflamed joints was also documented in PsA patients [29]. Compared with peripheral blood and synovial fluid from RA patients, a higher proportion of IL-17-producing ILC3s in the synovial fluid of PsA patients were CCR6^+^NKp44^+^, indicating ILC3s might migrate into the inflamed joints through CCR6 in PsA patients [29]. Furthermore, the frequency of circulating CCR6^+^NKp44^+^ILC3s was inversely correlated with PsA disease activity, supporting the hypothesis of circulating ILC3s’ migration into inflammatory tissue [29]. However, in a more recent study, peripheral ILC distribution in PsA patients was investigated and found circulating ILC3s were positively correlated with disease activity [46]. These discrepancies might be explained by the different markers defined for ILC3s and gating strategies used in these studies. Further analyses demonstrated that circulating ILC2s tend to be lower in PsA patients with high disease activity and the ratio of ILC2s to ILC3s was inversely correlated with disease activity and extra-articular manifestations, suggesting ILC2/3 ratio as a marker to distinguish between remission and active patients [46]. In addition, ILC2/3 ratio was also inversely correlated with imaging signs of inflammation and bone structural damage, indicating the imbalance of ILC homeostasis control disease activity in PsA [46].

Gut inflammation is common in SpA patients [47]. While very scarce in most other organs in homeostasis, ILC3s were abundant in the intestine and seem to be of vital importance for the containment of commensal flora in the gut lumen [48]. Considering the association of ILC3s with both SpA and inflammatory bowel diseases (IBD) has been established, the connection of ILCs especially ILC3s with enteropathic arthritis could be expected [49]. Triggianese et al. reported that circulating IL-17-producing ILC3s were significantly higher in enteropathic arthritis patients than IBD and healthy controls, suggesting ILC3s might be functionally distinct in IBD and enteropathic arthritis [50]. The exact role of ILCs in enteropathic arthritis needs to be further investigated.

## Therapeutic implication and future perspective

Clinical trials have shown the effectiveness of targeting IL-23/IL-17 axis and TNFα in the treatment of SpA and RA, respectively. Since ILCs were thought to be important source of IL-17 and other inflammatory cytokines, it would be feasible to speculate that ILCs could be targets for therapeutic interventions in inflammatory arthritis. Although ILC-specific therapies are still lacking, there has been some evidence that modulating ILC2 responses would be a promising way to promote resolution of arthritis. One example is that systemic administration of IL-33 at early stage of arthritis induction protected against the development of arthritis, accompanied by activation of ILC2s [36]. From another point of view, the plasticity of ILCs offers possibility to develop specific treatment that induces differentiation of proinflammatory ILC1s/ILC3s into non-inflammatory subsets.

## Conclusions

A growing body of evidence has emerged in recent years that ILCs have been implicated in the pathogenesis of inflammatory arthritis. However, knowledge of ILC development, differentiation, and plasticity as well as truly specific markers in human is still limited, making it a great challenge to decipher the exact role of ILCs in the context of inflammatory arthritis. Future work should focus on the regulation pathways of ILCs in different environmental milieus, which would be helpful to get better understanding of ILCs in arthritis initiation, progression, and resolution. These efforts will ultimately aid in the design of effective strategies for controlling arthritis.

## Data Availability

Not applicable
